# Potential Toxicity of Microplastics in Wheat and Maize—A Pilot Pot Experiment

**DOI:** 10.3390/plants15193043

**Published:** 2026-10-05

**Authors:** Slaveya Petrova, Bogdan Nikolov, Petar Mandaliev, Georgi Stanchev, Stela Stoyanova, Elenka Georgieva, Veska Kashorova, Valentina Raykova, Plamen Marinov-Serafimov, Irena Golubinova

**Affiliations:** 1 Department of Ecology and Environmental Conservation, Faculty of Biology, Paisii Hilendarski University of Plovdiv, 24 Tsar Asen Street, 4000 Plovdiv, Bulgaria; nikolov81bg@uni-plovdiv.bg (B.N.); vkashorova@uni-plovdiv.bg (V.K.); stu2504758003@uni-plovdiv.bg (V.R.); 2 Department of Microbiology and Ecological Biotechnologies, Faculty of Plant Protection and Agroecology, Agricultural University, 12 Mendeleev Boulevard, 4000 Plovdiv, Bulgaria; 3 Centre for Cleantech and Biomass Resource Efficiency, Agricultural University, 12 Mendeleev Boulevard, 4000 Plovdiv, Bulgaria; petar.mandaliev@fhnw.ch; 4 Institute for Biomass and Resource Efficiency, University of Applied Sciences Northwestern Switzerland, 5210 Klosterzelgstrasse 2, 5210 Windisch, Switzerland; 5 Department of Crop Production, Faculty of Agronomy, Agricultural University, 12 Mendeleev Boulevard, 4000 Plovdiv, Bulgaria; joro_stanchev@abv.bg; 6 Department of Developmental Biology, Faculty of Biology, Paisii Hilendarski University of Plovdiv, 24 Tsar Asen Street, 4000 Plovdiv, Bulgaria; stela.stoyanova@uni-plovdiv.bg (S.S.); elenkageorgieva@uni-plovdiv.bg (E.G.); 7 Institute of Ornamental and Medicinal Plants, Agricultural Academy, 1222 Sofia, Bulgaria; plserafimov@abv.bg (P.M.-S.); golubinova@abv.bg (I.G.)

**Keywords:** polyethylene, soil pollution, photosynthesis, transpiration, growth, seed germination

## Abstract

Most agricultural plastic products are single-use and often remain in the environment long after they have fulfilled their intended purpose. From the soil, they may transfer to living organisms, where they can alter biological functions, physiological processes, and reproduction, and can also lead to pollutant accumulation in biomass; through food chains, they may increase risks to human health. This study analyzes the uptake of polyethylene microplastics (MPs) in wheat and maize, and their impact on plant development. Two types of fluorescent polyethylene spheres were used in a pot experiment—27–32 µm and 75–90 µm in diameter, as these sizes are reported to penetrate plant root tissues most efficiently. After 50 days, MPs accumulated in all organs of the test plants—roots, stem, and leaves. The average relative growth rate (RGR) shows that the two plant species respond differently to the applied microplastics. In maize, the control plants recorded the highest RGR (0.039 day^−1^), while both MP treatments decreased RGR (0.034 day^−1^ for 75–90 µm and 0.026 day^−1^ for 27–32 µm). In wheat, the lowest RGR was recorded under 75–90 µm MPs exposure (0.016 day^−1^), while at 27–32 µm MPs the RGR value (0.024 day^−1^) practically coincides with that of the control plants. MPs significantly affected growth and biomass synthesis in both underground and aboveground organs, with the degree of impact depending on plant type and MP size. Polyethylene microplastics positively affect photosynthetic pigment biosynthesis in maize and negatively affect it in wheat. Future comprehensive studies in real field conditions are needed to reveal the impact of MPs on plants and their ability to be transmitted through food chains, thus assessing the risk to food security and human health.

## 1. Introduction

Because of their widespread use, low cost, physicochemical properties, durability, and ease of production, plastics are found practically everywhere around us [1]. Their slow degradation in nature makes them a major pollutant in ecosystems worldwide [2]. Their degradation involves gradual fragmentation into smaller pieces, namely microplastics (MPs) and nanoplastics (NPs) [3]. Because of excessive use and improper waste management, MPs and NPs enter the world’s oceans, soils, air, and living organisms, posing a serious threat to their well-being and survival [4]. Microplastics and nanoplastics penetrate all food chains [5,6]. Studies show that people ingest significant amounts of microplastics daily through food, especially seafood, and through inhalation, with estimates ranging from 39,000 to 52,000 particles per person per year [7,8]. Furthermore, MPs in human feces suggest widespread exposure and absorption [8].

MPs in terrestrial ecosystems are still poorly understood, and their effects on soil and biota are not fully known. Research on plastic pollution in soil began in 2012 and has increased in recent years [9]. In general, microplastics in the soil are difficult to see with the naked eye because the two components are well homogenized. Plastic pollution in soil is dangerous because wind can carry it, plastics degrade very slowly, and they may have toxic effects on soil organisms [10,11,12]. Some studies show that when earthworms ingest microplastics, they suffer inflammation and digestive problems that affect their development and reproduction [13]. Similar problems have also been found in single-celled soil organisms, which can prevent the maintenance of healthy and favorable soil [14]. Microplastics can also physically disrupt soil, as fibers and fragments alter soil aggregation, compaction, and cohesion [15]. Microplastics can also contaminate soil with chemicals introduced during pretreatment to achieve desired product qualities. Over time, these chemicals can leach into the soil and affect soil microorganism development and function [15,16,17,18].

The pathways for microplastics to enter the soil are extremely diverse, including plastic bottles or bags that degrade slowly in the soil, household laundry wastewater, cleaning agents and cosmetics, and many others [19]. Wastewater treatment plants capture some of the plastic in the water, but another portion cannot be captured and goes to rivers, from where it can pass into other components of the environment [20,21,22]. The use of plastic products in modern agriculture is increasingly common worldwide, as it contributes to higher yields, reduced water and herbicide use, and a controlled increase in crop quality at low cost, but with a high risk of an impending ecological crisis [23,24,25,26,27]. Most agricultural plastic products are single-use and often remain in the field long after they have fulfilled their intended purpose [5]. In recent years, it has been shown that soil-derived microplastics penetrate seeds, roots, stems, leaves, fruits, and plant cells, and the ability for translocation and bioaccumulation depends mainly on their size and type [28,29]. MPs disrupt biological functions and physiological processes, including reproduction, and lead to the accumulation of pollutants in biomass, which, through food chains, inevitably increases the risk to human health [11,30,31,32]. The detection of MP absorption and translocation within plants currently faces a multitude of challenges and complexities, and the use of fluorescently labeled plastics to treat plants, followed by microscopic observation of plant tissues, can be very useful [33].

Wheat and maize are among the most economically important crops worldwide, which is why they are often studied in relation to the uptake of microplastics and their impact on plant development [34,35,36]. For example, Feng et al. [34] found that polyethylene MPs (10 μm and 500 μm) exerted significant stress on the growth and development of winter wheat, expressed as “low promotion (0.1–0.5%) and high inhibition (1.0–5.0%)”. This was accompanied by increased soil enzyme activity (soil urease, soil acid phosphatase, and soil dehydrogenase) in winter wheat. Ren et al. [35] reported that 5 μm polystyrene beads had a lower inhibitory effect on biomass synthesis (2–3%) in wheat (both belowground and aboveground) than on plant height (12%) and base diameter (29%) of seedlings after a 2-week pot experiment. Similarly, Zhang et al. [37] conducted experiments with three microplastic types (polypropylene, high-density polyethylene, and polylactic acid), three particle sizes (150, 1000, and 4000 μm), and three concentrations (0.1, 0.5, and 1 g·kg^−1^) to study their impact on wheat growth and rhizosphere soil environment. They concluded that the effects of MPs on wheat seed growth characteristics were inconsistent, but polylactic acid MPs had the most pronounced inhibitory effect overall. Regarding the impact of MPs on maize, Sun et al. [36] found a dose-dependent relationship, with stronger inhibition of plant growth and performance at 10% polyethylene in soil. Usman et al. [38] concluded that polyethylene MPs significantly inhibited maize growth, reducing root fresh weight by up to 32% at high doses and small particle sizes (<0.212 µm). However, none of these, and only a few other existing literature sources in the field, provided evidence from microscopic observations of bioaccumulated MPs in wheat [37] and maize [39] tissues.

All recent reports highlight the potential threat of microplastics to agricultural productivity, the need for further research into their mechanisms of action and mitigation strategies in agroecosystems, and the risk they pose to food chains and food security. The present study analyzes the uptake of polyethylene microplastics in wheat and maize, as well as their impact on plant development. Two types of fluorescent polyethylene spheres were used in a pot experiment—27–32 µm and 75–90 µm—as these sizes are reported to penetrate plant root tissues most efficiently. Bioaccumulation of MPs in both below- and aboveground plant parts was identified by fluorescent microscopy, a fast, sensitive, cost-effective screening method that is still underestimated. The effects on seed germination, leaf traits, and plant development were assessed using various statistical analyses to evaluate the significance of differences among treatments.

## 2. Results

### 2.1. Effect on Seed Germination

**Figure 1:**
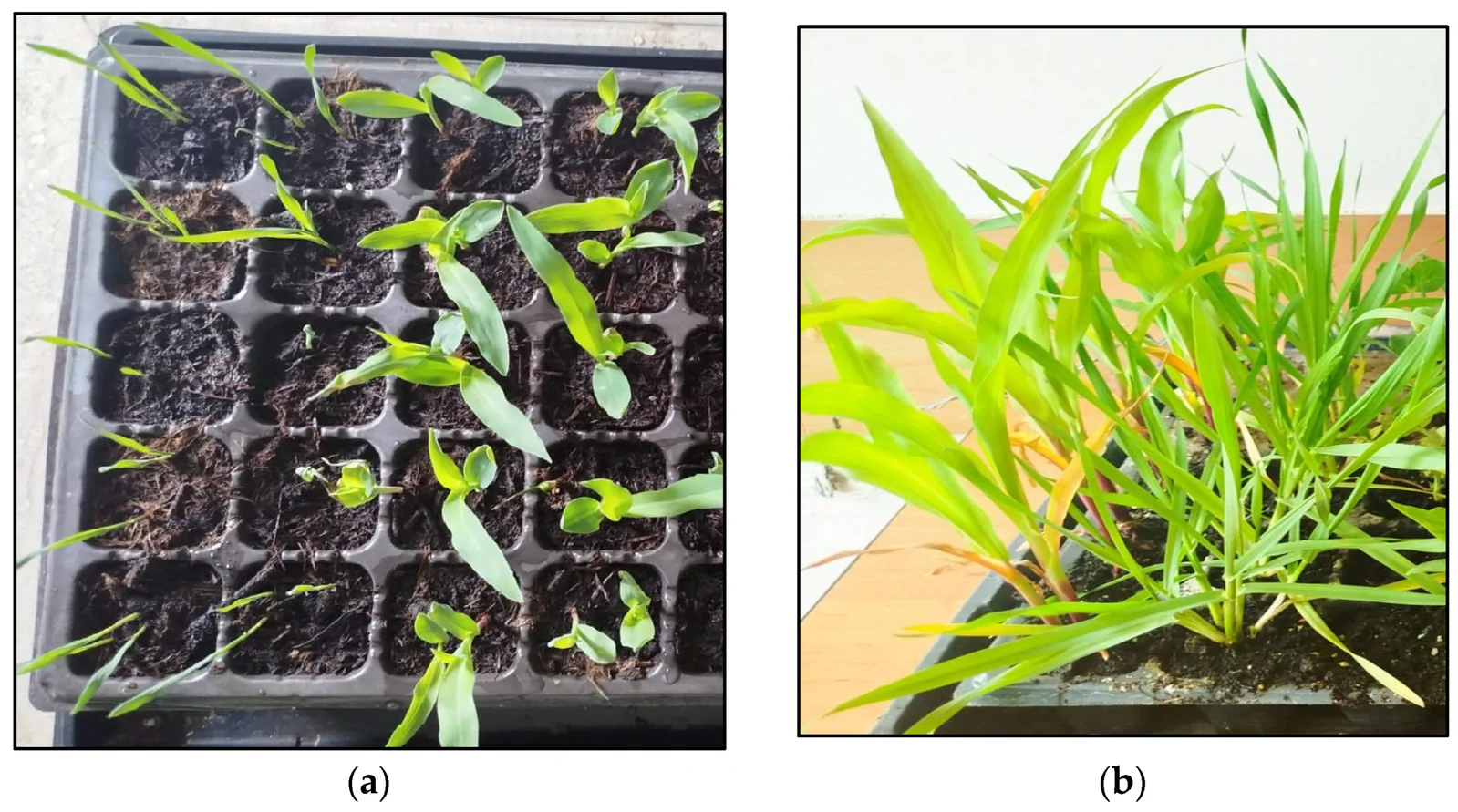
Seed germination and plant development under MP exposure—control without MPs is on the top (row 1), 27–32 µm MPs are in the center (rows 2 and 3), 75–90 µm MPs are on the bottom (rows 4 and 5); (**a**) Day 7 (3 April 2025); (**b**) Day 50 (16 May 2025).

Biotests with fluorescent polyethylene MPs were set up on 27 March 2025, and on the 7th day (2 April 2025), 100% germination was observed in both the controls and all experimental treatments (Figure 1). Therefore, MPs had no impact on the germination of maize and wheat seeds during the experiment. These results align with other authors’ findings, showing that exposure to microplastics for up to 1 day had a suppressive effect on germination. However, exposure for 2–4 days or more no longer significantly affected seed germination [14,17,18,24,34,35,40]. This may be explained by certain plant species’ ability to adapt to microplastic particles after a certain exposure time. Studies also report improved seed germination at higher microplastic concentrations [41,42]. For instance, Ge et al. [43] indicate that variations in seed germination may stem from the seeds’ ability to adsorb microplastics—a process dependent on the charge, particle size, and agglomeration of microplastics in the soil—while also noting the need to consider the specific characteristics of each plant species. It is also evident that seeds of different plant species respond differently to microplastic contamination in terms of germination parameters; therefore, more research is needed to investigate this relationship.

### 2.2. Effect on Plant Growth

**Figure 2:**
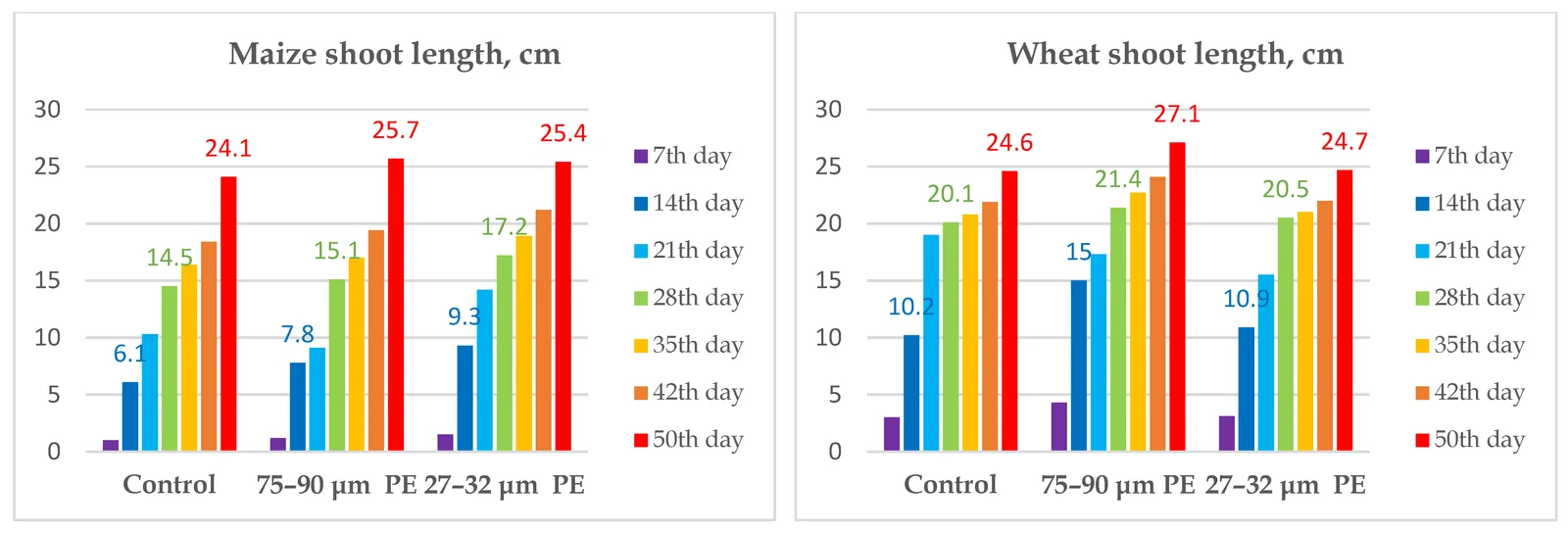
Plant growth during the experiment with MPs.

Observations on the growth of the experimental plants were conducted periodically, with the length of the above-ground organs measured once per day. Biometric measurements of shoot length (cm) allow an objective assessment of the effects of microplastics on the initial stages of development in both crops. By the 14th day (Figure 2), control plants had smaller shoots than those under MP exposure. Over the next few days, the data fluctuated, but by the 28th day the trend reappeared and persisted until the final 50th day of the experiment. The results confirm that seed germination is a less sensitive stage in ontogenesis, whereas shoot growth can be used to assess the impact of microplastics on plants.

The length of the maize shoot ranged from 24.1 cm (control) to 25.7 cm (75–90 μm MPs), and the root length ranged from 9.1 cm (75–90 μm MPs) to 19.7 cm (27–32 μm MPs), with significant differences compared to the control (*p* < 0.05) (Figure 2). In wheat, the shoot length ranged from 24.6 cm (control) to 27.1 cm (75–90 μm MPs), and the root length ranged from 9.4 cm (control) to 13.5 cm (27–32 μm MPs), with significant differences also observed (*p* < 0.05) (Figure 2).

To quantitatively assess growth dynamics over the entire experimental period, the average relative growth rate (Relative Growth Rate, RGR) was calculated according to the methodology described by Hunt [44] and Hunt et al. [45]. The indicator characterizes the relative growth of plants per unit time and was determined by the formula:

$$\mathrm{RGR}=\frac{\ln(H_{2})-\ln(H_{1})}{t_{2}-t_{1}}$$

where RGR—relative growth rate (day^−1^),

*H*_1_ and *H*_2_—average length of aboveground organs (cm) at two consecutive measurements;

*t*_1_ and *t*_2_—time (days) of the first and second measurement;

ln—natural logarithm.

The average relative growth rate (RGR) shows that the two plant species respond differently to the applied microplastics (Table 1). In maize, the control plants had the highest average RGR (0.039 day^−1^), while both MP treatments decreased RGR. A slight decrease was found with 75–90 µm particles (average RGR = 0.034 day^−1^), while 27–32 µm particles produced the lowest relative growth rate (average RGR = 0.026 day^−1^), indicating a downward trend. In wheat, the lowest average RGR was recorded in plants treated with 75–90 µm MPs (0.016 day^−1^), while at 27–32 µm MPs the average RGR (0.024 day^−1^) nearly coincided with that of the control plants. These results suggest that smaller polyethylene particles did not significantly affect the relative growth rate of wheat during the experimental period.

The results obtained show a species-specific response to MPs that depends on particle size. In maize, both sizes were associated with lower RGR values than the control, with a more pronounced trend for particles with a size of 27–32 µm. In wheat, RGR decreased only in plants exposed to 75–90 µm, while smaller particles produced values comparable to those of the control plants.

The results obtained at the end of the experiment (Table 2) show that the impact of polyethylene MPs depends on both particle size and plant species. Statistically significant differences between treatments were found at *p* ≤ 0.05 and are indicated by different letters. The biomass of the maize shoot ranged from 1.88 g (27–32 μm MPs) to 1.98 g (control), while the root biomass of plants under 27–32 μm MPs exposure was significantly different from the control and from that under the larger polyethylene MPs (*p* < 0.05) (Table 3). The biomass of the wheat shoot varied from 0.19 g (27–32 μm MPs) to 0.34 g (control), and that of the roots varied from 0.11 g (27–32 μm MPs) to 0.25 g (75–90 μm MPs), with reliable differences demonstrated only for the smaller MPs (Table 2).

In maize, no significant differences were found in aboveground length, but belowground length was largest under 75–90 µm MPs exposure and smallest under 27–32 µm MPS exposure. Both MP sizes significantly decreased belowground biomass compared to the control, and for aboveground biomass, the most significant negative effect was observed under 75–90 µm MPs.

In wheat, both MP sizes caused a statistically significant increase in aboveground length compared to the control. Root length increased only under 75–90 µm MP exposure. Aboveground biomass was statistically significantly lower in both MP treatments, while belowground biomass showed a different effect—a decrease at 75–90 µm and a statistically significant increase at 27–32 µm.

The results show a clear species-specific response to polyethylene MPs, with particle size having a significant impact on growth and biomass accumulation in both crops.

### 2.3. Effect on Plant Physiology

**Figure 3:**
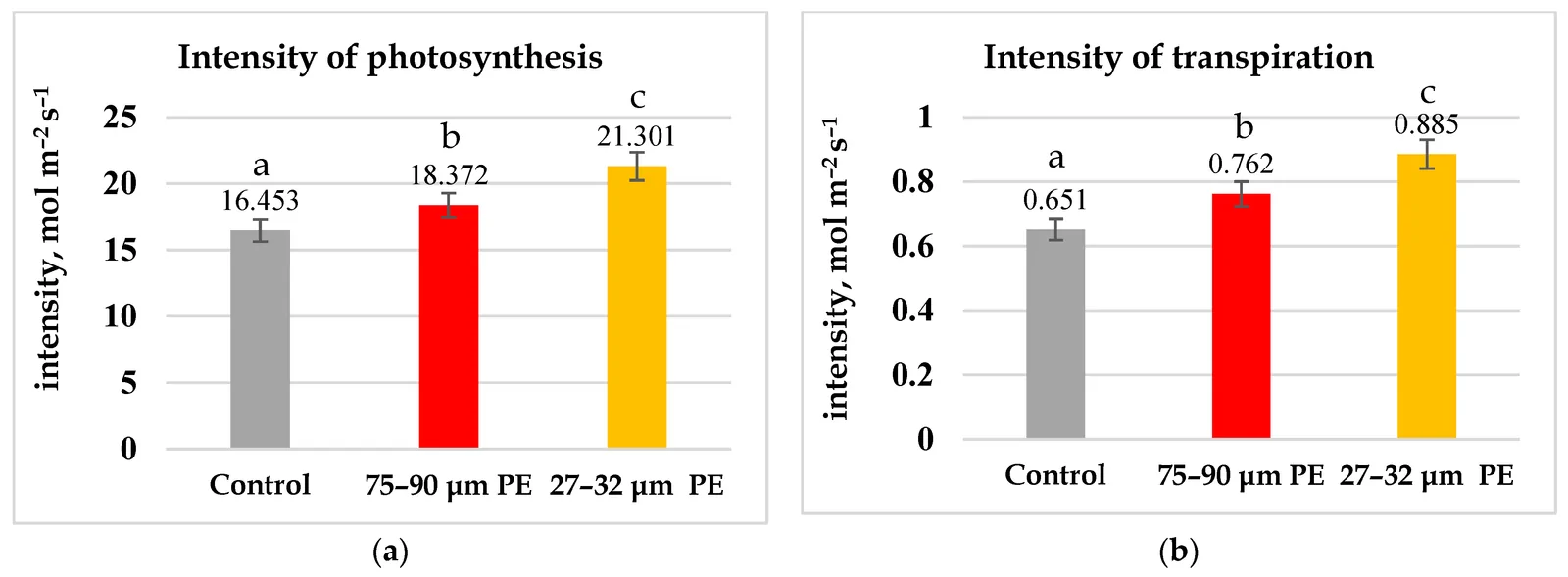
Physiological properties of studied maize plants under MPs exposure on the 50th day of the experiment: (**a**) photosynthesis intensity; (**b**) transpiration intensity. Statistically significant differences between treatments were found at *p* ≤ 0.05 and are indicated by different letters.

**Figure 4:**
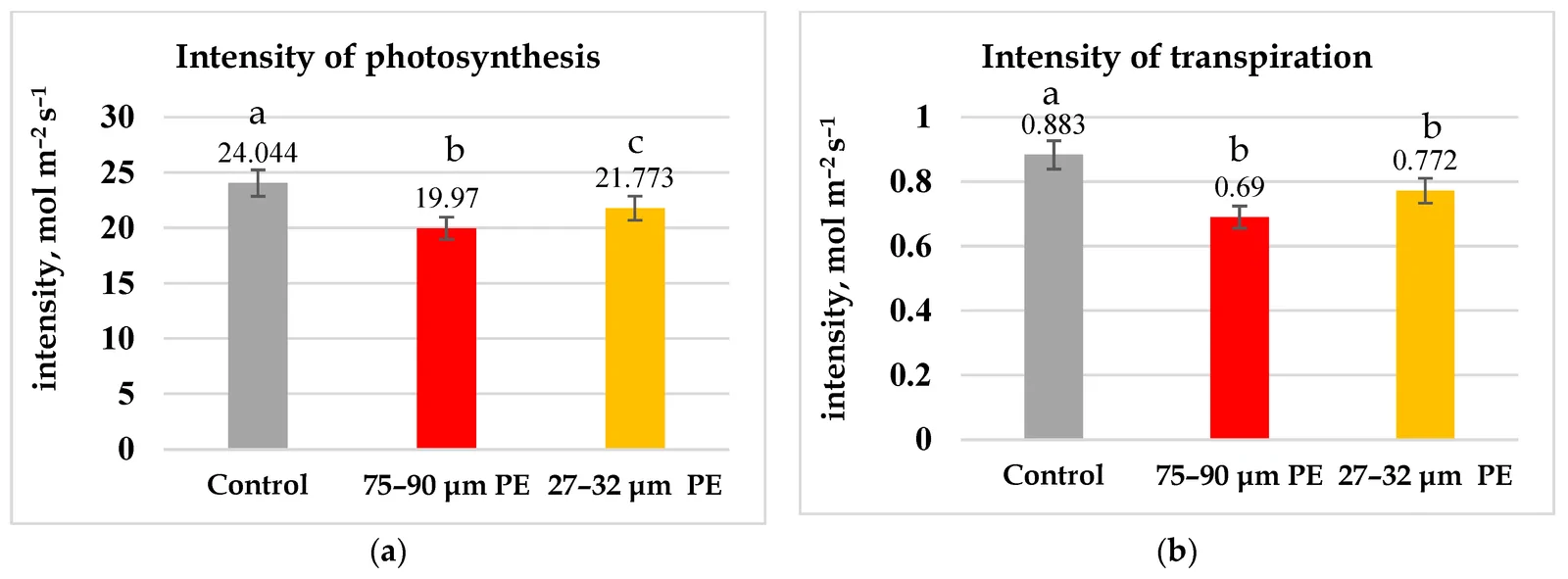
Physiological properties of studied wheat plants under MPs exposure on the 50th day of the experiment: (**a**) photosynthesis intensity; (**b**) transpiration intensity. Statistically significant differences between treatments were found at *p* ≤ 0.05 and are indicated by different letters.

Polyethylene microplastics affect physiological processes in maize, as shown by increased photosynthesis (Figure 3a) and transpiration (Figure 3b) intensity, with a stronger effect in plants treated with smaller MPs (27–32 μm). An opposite trend is observed in wheat plants—a suppression of the two fundamental processes in the presence of MPs, which is more pronounced in plants treated with the larger MPs (75–90 μm) (Figure 4).

**Figure 5:**
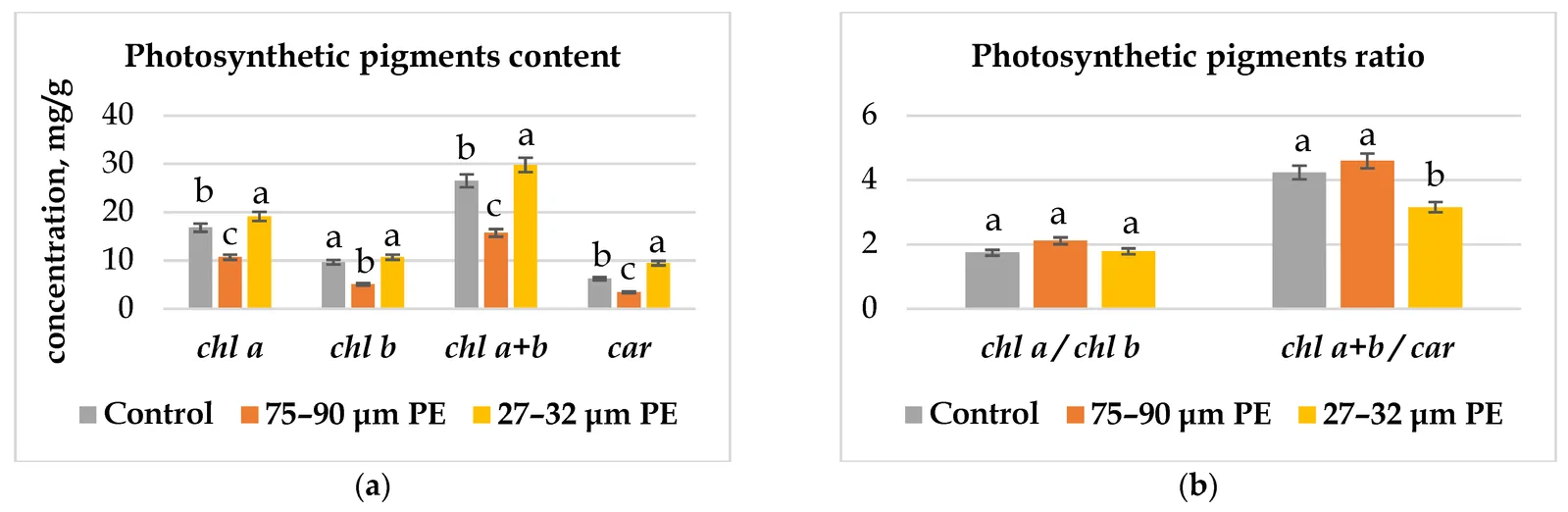
Pigments in maize leaves under MPs exposure on the 50th day of the experiment: (**a**) content; (**b**) ratios. Statistically significant differences between treatments were found at *p* ≤ 0.05 and are indicated by different letters.

**Figure 6:**
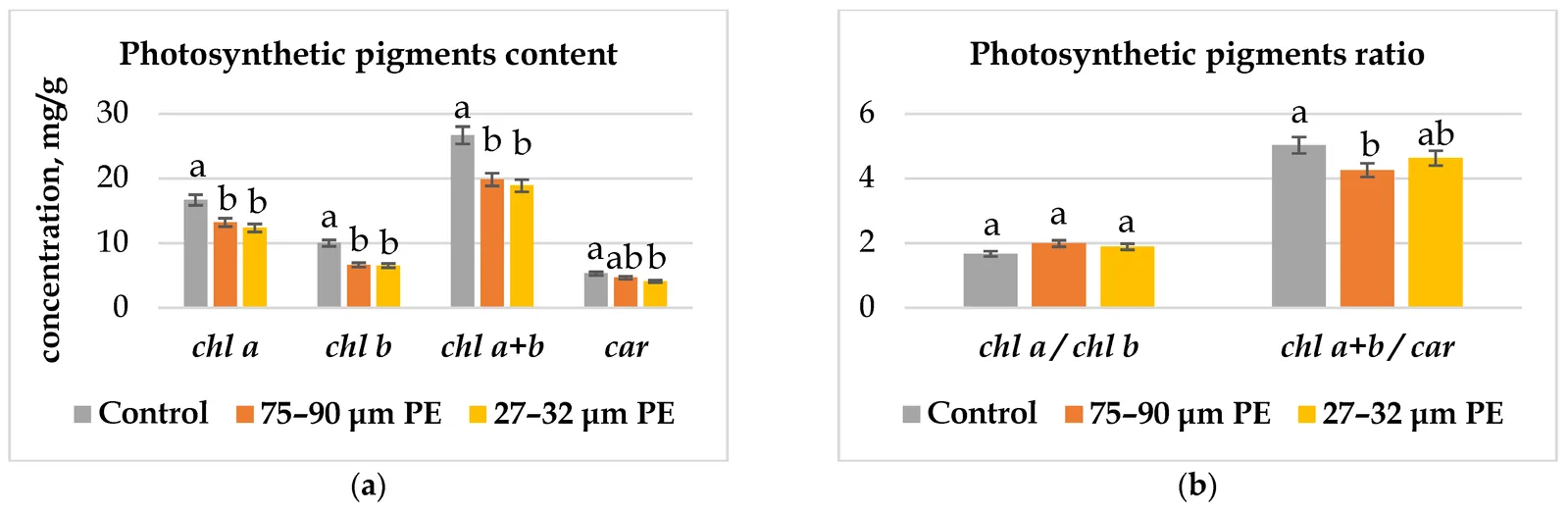
Pigments in wheat leaves under MPs exposure on the 50th day of the experiment: (**a**) content; (**b**) ratios. Statistically significant differences between treatments were found at *p* ≤ 0.05 and are indicated by different letters.

Smaller MPs (27–32 μm) significantly increased chlorophyll and carotenoid levels in maize leaves, while larger MPs (75–90 μm) suppressed pigment synthesis (*p* < 0.05). However, the ratios between the two chlorophyll types remained almost unchanged compared with the control. In contrast, the ratio between chlorophylls and carotenoids showed a clear increase (Figure 5). In wheat, a suppressive effect on the photosynthetic pigment content was observed in the presence of both types of MPs, as well as a decrease in the values of the ratio between chlorophylls and carotenoids (Figure 6).

### 2.4. Bioaccumulation of MPs in Plants

**Figure 7:**
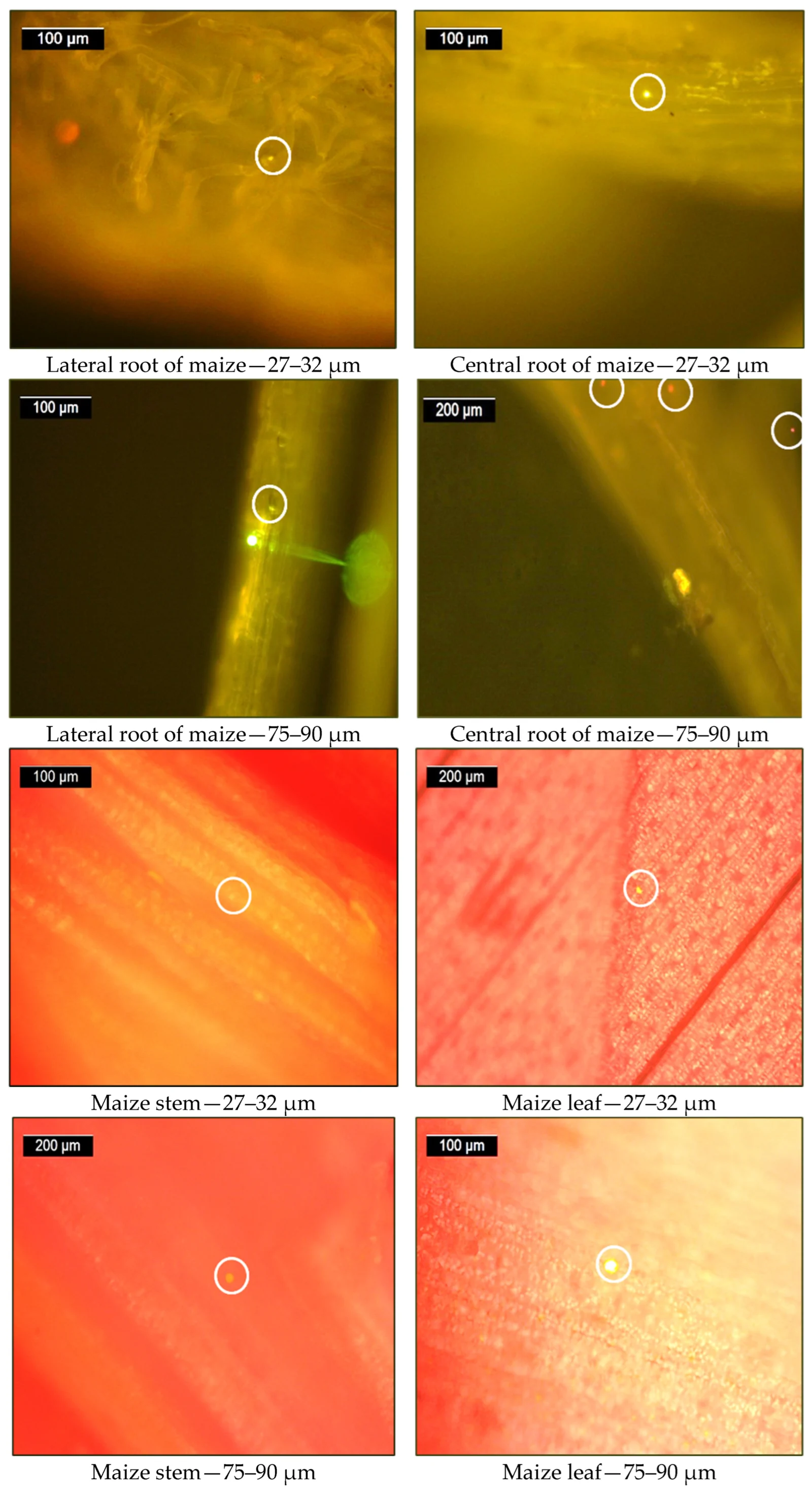
Bioaccumulation of fluorescent MPs in maize—roots, stem, leaves. MPs found in plant organs are surrounded by a circle.

**Figure 8:**
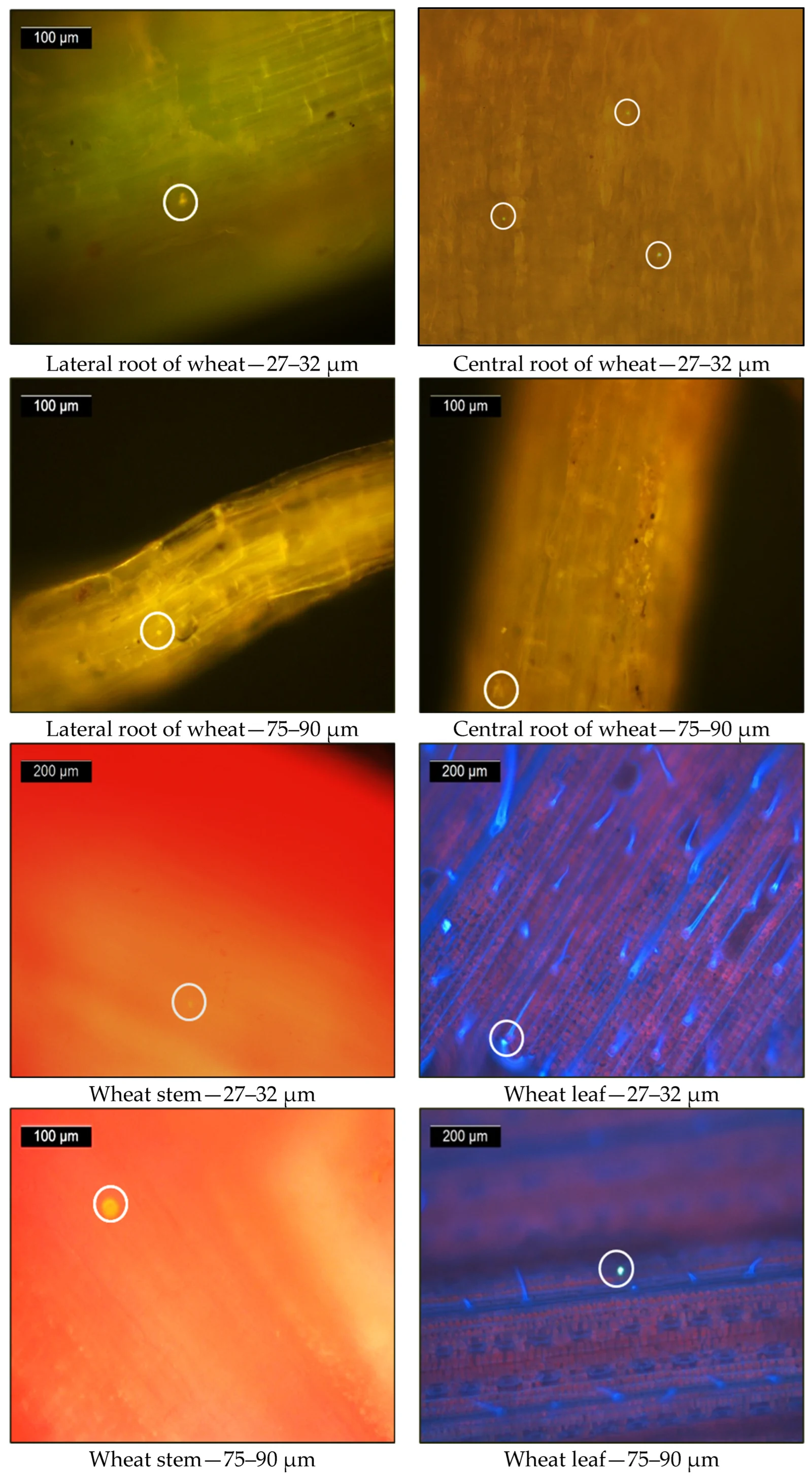
Bioaccumulation of fluorescent MPs in wheat—root, stem, leaves. MPs found in plant organs are surrounded by a circle.

The bioaccumulation of MPs in maize (Figure 7) and wheat (Figure 8) was observed using a fluorescence microscope on thin slices from the underground and above-ground plant organs: central root, lateral roots, stem, and leaves. Polyethylene MPs of both studied sizes (27–32 μm and 75–90 μm) were found in all organs of the experimental plants. In general, the smaller MPs showed the highest number of microplastic particles per visual field, and higher MP levels were found in the root system than in the shoot (stem and leaves). Calculated median values (per area) of 27–32 μm MPs, detected in plant organs, are as follows: lateral root—26 items cm^−1^ (maize) and 19 items cm^−1^ (wheat), central root—37 items cm^−1^ (maize) and 31 items cm^−1^ (wheat), stem—20 items cm^−1^ (maize) and 17 items cm^−1^ (wheat), leaves—14 items cm^−1^ (maize) and 8 items cm^−1^ (wheat). Calculated median values (per area) of 75–90 μm MPs, detected in plant organs, are as follows: lateral root—16 items cm^−1^ (maize) and 11 items cm^−1^ (wheat), central root—22 items cm^−1^ (maize) and 17 items cm^−1^ (wheat), stem—18 items cm^−1^ (maize) and 9 items cm^−1^ (wheat), leaves—11 items cm^−1^ (maize) and 6 items cm^−1^ (wheat). The presence of MPs in plant tissues and organs may be associated with observed disturbances in metabolism, biosynthesis of key substances, and plant functioning. They, in turn, affect the growth and development of the test plants observed during the experiment. Similar experiments should be conducted under laboratory and field conditions to track the overall manifestation of these effects through the full vegetative development cycle.

### 2.5. Effect on Soil Properties

Table 3 shows the effects of two types of polyethylene MPs on soil physicochemical properties, along with the ranges of increase or decrease in their average values under various treatments compared with the control. Total nitrogen (TN) concentration differed significantly between the MPs treatment and control pots, with the largest differences observed in wheat with 27–32 µm PE. Reduction rates ranged from 8% to 14%, indicating that polyethylene MPs significantly affect the reduction in TN in rhizosphere soil. A reverse effect was found for total phosphorus (TP), with an increase (*p* < 0.05) ranging from 2% (wheat) to 6.5% (maize). These results align to some extent with Zhang et al. [37], who also observed the same trends. Total potassium (Total K) concentrations did not differ significantly between the MPs treatment and the control, although a slight decrease occurred. OM concentrations also did not differ significantly, and the decrease was too small. Conductivity also showed a decreasing trend with no statistical significance, and it was more visible in wheat pots. pH values showed a slight decreasing trend across different particle sizes compared with the control, but the differences were not significant.

## 3. Discussion

Seed germination is the transition of plant seeds from dormancy to active life, the initial stage of plant ontogenesis, in which a sprout is formed. It occurs in the presence of moisture and oxygen, appropriate temperatures, and light. During germination, metabolism in the embryo and endosperm increases; seeds swell in water, and starch, fats, and proteins break down into sugars, fatty acids, and amino acids. Usually, the root germinates first, followed by the hypocotyl or epicotyl (depending on the plant). Seed germination is a complex process involving several signals and influenced by both internal and external factors. Seed germination is very often used as an indicator for toxicity assessment in various bioassays, as it allows determination of the biological activity of various substances (herbicides, metabolites, allelochemicals and even radionuclides) with high reliability and low cost [46,47]. Our results showing no effect of MPs on seed germination in wheat and maize agree with Zhang et al. [37] and Fajrado et al. [48,49,50,51,52,53,54], who also used polyethylene in their experiments. Sahasa et al. [49] found that exposure to different concentrations of microplastics significantly reduced seed germination in black bean and tomato during the first 24 h of exposure. However, after 48 h, germination reached over 90% in all treatments, regardless of the concentration of microplastics. Furthermore, with increasing incubation time (i.e., after 72 h), germination did not change, which correlates well with our data. Pehlivan & Gedik [50] reported some inhibition of maize seed germination under PVC exposure. Other studies report improved seed germination at higher microplastic concentrations [42,47]. For example, Ge et al. [43] suggest that variation in seed germination may reflect seeds’ ability to adsorb microplastics, which depends on the charge, particle size, and agglomeration of microplastics in the soil; species-specific characteristics must also be considered.

One of the most visible effects of microplastics on plants is altered growth, ranging from inhibition to indifference to stimulation (Table 4). In Fajardo et al. [48], maize plants showed reduced root and shoot lengths after polyethylene contamination. Still, maize plants in an experiment with polyurethane-coated manure were not affected [47]. Our study recorded reduced shoot and root length in maize under 27–32 µm MPs exposure, but increased length under 27–32 µm MPs exposure (Table 2). Nevertheless, biomass was significantly lower than in the control (*p* < 0.05). A similar trend was observed for wheat biomass, while the stimulating effect on plant growth was pronounced (*p* < 0.05). Our results agree with other authors who reported that 200 μm polyethylene microplastics at a concentration of 0.2% in soil stimulated wheat plants over 42 days [14]. Low-density polyethylene particles (200–250 μm) inhibited leaf stalk length in wheat varieties but stimulated it in a short-term experiment (15 days) [49]. The same microplastics did not affect wheat growth in a long-term experiment conducted by Qi et al. [51]. In laboratory experiments to determine the impact of different types of microplastics on watercress for a period of 7 days and for a period of 21 days, Pignatelli et al. [18] also demonstrated a lack of effect on seed germination, as well as a stimulating effect on the growth and accumulation of biomass in the above-ground part of the plants.

Photosynthesis is a fundamental, yet highly sensitive process in plants; therefore, it is often used as an early indicator of stress. The content of photosynthetic pigments and the ratios among different pigment functional groups are also important. Photosynthesis is a good indicator of plant sensitivity to stress conditions, because maintaining high photosynthetic activity is extremely important in antioxidant protection and recovery mechanisms. Transpiration is a fundamental process in the water regime of plants, since it creates an energy gradient and acts as the driving force of sap flow in a plant. In general, transpiration is more intense in young, healthy plants than in old or stressed plants. The plant response to this stress factor at the organism level is complex because it reflects the integration of stress effects and the physiological responses of lower structural-functional levels of organization across time and space. In our 50-day experiment, we observed two contrasting responses to MPs exposure—enhanced photosynthesis in maize (Figure 3) and inhibited photosynthesis in wheat (Figure 3). There are various results in the literature; for example, Dong et al. [55] investigated the effects of polystyrene microplastic granules on the growth and quality of red lettuce, also finding bioaccumulation of microplastics in the root system. Similar to our results, they found that the effects on plant physiology were mainly expressed as decreased chlorophyll content, photosynthesis and transpiration rates, and soluble sugars and proteins (Table 4).

Meng et al. [56] reported that low-density polyethylene (250 μm–1000 μm) in soil reduced chlorophyll content in common bean leaves. At the same time, the same size and amount of biodegradable microplastics had a stimulating effect. PE and PLA microplastics did not affect photosynthetic pigments in maize [26], but PVC microplastics significantly suppressed them [57], and polyurethane-coated fertilizer significantly stimulated them [47]. Chlorophyll content in wheat was unchanged or even stimulated by low doses of PE (up to 1% *w*/*w*), but was significantly impaired at higher concentrations [14,51,52]. In the present study, MPs of the smaller size (27–32 μm) significantly increased the amount of chlorophyll and carotenoids in maize leaves, while the opposite effect in wheat was pronounced. Li et al. [58] conducted similar studies using cucumber as the test object and polystyrene nanoplastic granules (sizes 100 nm, 300 nm, 500 nm, 700 nm). The authors found a significant decrease in the length and biomass of the underground and aboveground parts of the treated plants only at 300 nm, concluding that plastic particle size is decisive for the degree of impact. This also implies the observed changes in photosynthetic intensity and the decrease in photosynthetic pigment content depending on nanoplastic size [50,52].

Our study has some limitations that do not decrease its scientific soundness. (1) The experiment was performed in greenhouse conditions that could not fully represent the natural environment. However, we aimed to assess the potential of two widely used crops (wheat and maize) to accumulate MPs in their tissues, and we successfully did so. (2) The observations on MP accumulation were made by fluorescence microscopy, which cannot provide detailed data on the quantity of microplastics in plant tissue. Nevertheless, this method allows for fast and inexpensive screening of fluorescent MPs in various plant organs.

Future complex studies are necessary for a more complete disclosure of the impact of microplastics on plants and their ability to be transmitted through food chains, reaching humans.

## 4. Materials and Methods

### 4.1. Test Materials—Soil, Seed and MPs

The experiments used standardized soil (substrate) purchased from a certified manufacturer (Agro CS, Rikov, Czech Republic). Soil sample preparation for the analysis of physico-chemical parameters was carried out in the Agroecology laboratory (Faculty of Plant Protection and Agroecology, Agricultural University-Plovdiv) according to ISO 11464 [59], and the following analyses were performed: organic carbon, organic matter (calculation method), total nitrogen (BDS ISO 11261:2002 [60]), total phosphorus, and total potassium (GOST 26209:1991 [61]). Soil pH was measured in distilled water using pHotoFlex Set–Model 2512000, WTW-Germany (ISO 10390 [62]), and soil conductivity was measured using Multiset, F340, WTW-Germany (ISO 11265 [63]). Soil moisture was determined using the weight method. The initial values of the main soil characteristics are presented in Table 5.

The biotests used wheat (*Triticum aestivum* L.) and maize (*Zea mays* L.) seeds, purchased from a certified manufacturer. These two plant species were selected because they are among the most widely grown crops worldwide and are widely used as a food resource for humans and farm animals. All seeds were surface-sterilized by soaking them in a 1% sodium hypochlorite solution for 10 minutes, then washed three times with distilled water. Excess water on the surfaces of seeds was absorbed by filter paper before sowing the seeds in pots.

**Figure 9:**
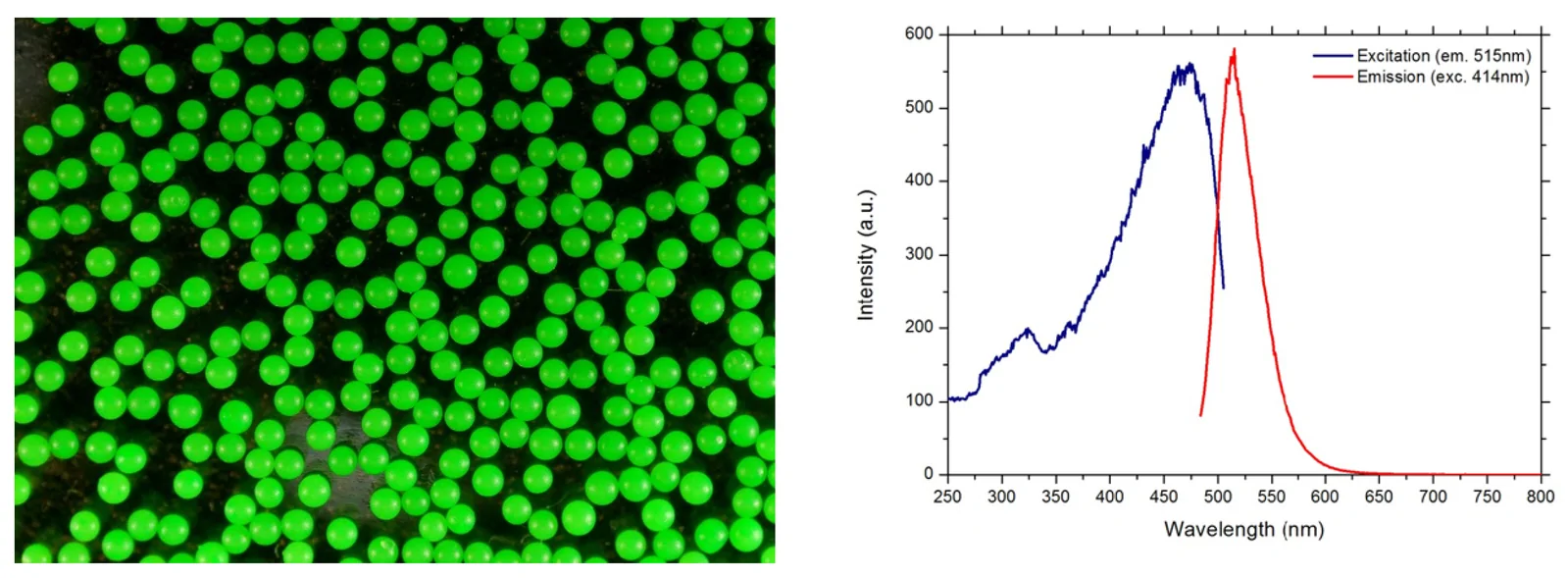
Main properties and shape of polyethylene MPs used in the experiments. [Source: https://www.cospheric.com/UVPMSBG_fluorescent_green_spheres_density1025htm, Accessed on 25 March 2025].

The experiments used polyethylene MPs in two sizes, colored with a fluorescent green pigment, as follows: Fluorescent Green Polyethylene Microspheres 27–32 µm diameter and Fluorescent Green Polyethylene Microspheres 75–90 µm diameter (Cospheric, Somis, CA, USA). Figure 9 shows the shape and spectral response curve provided by the manufacturer.

### 4.2. Experimental Setup

The biotesting was conducted under controlled conditions in a greenhouse at the Agricultural University of Plovdiv, using 0.1-L pots. Each pot was filled to 2/3 of its volume with standardized soil, to which the selected MP types were added at a concentration of 0.01% *w*/*w* (1 mg MPs per 100 g of soil) based on previous studies [64,65]. Two seeds of maize or wheat were placed in each pot, and a 1.5 cm soil layer was added on top [64,65]. The controls were set up in the same way, but without adding microplastics to the soil.

**Figure 10:**
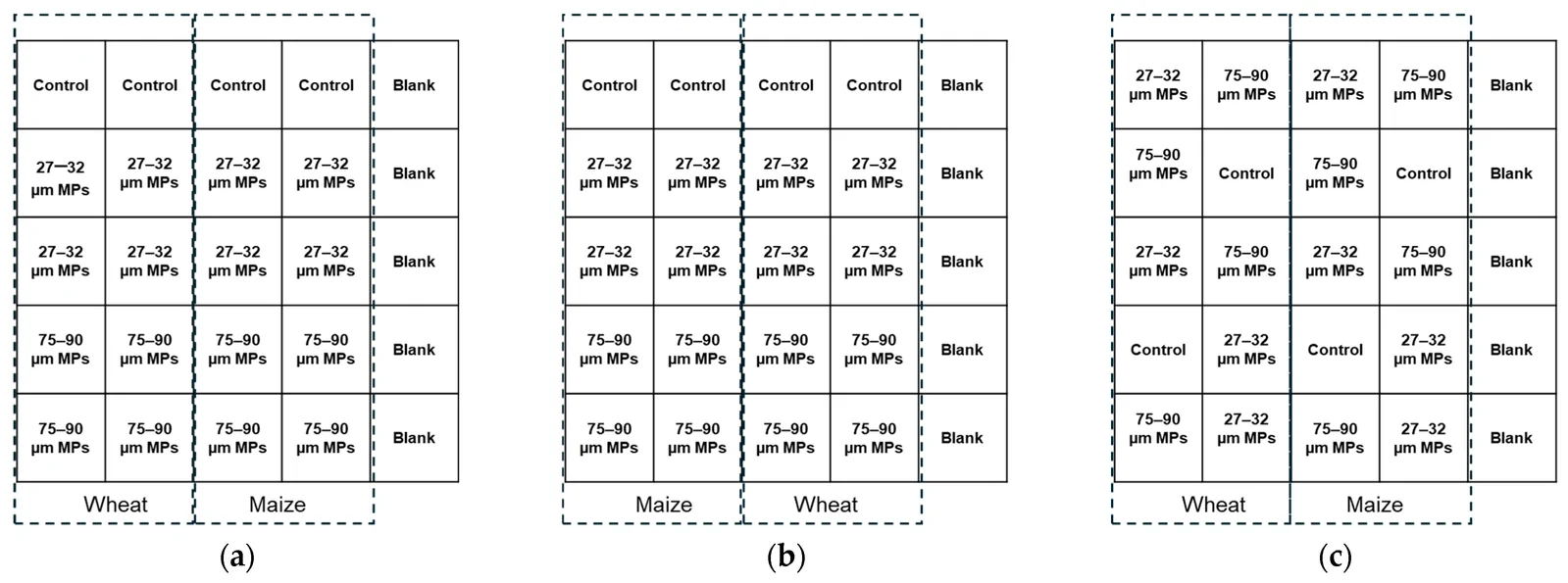
Schematic representation of the experiment, including three trays (**a**–**c**), with a total of 6 control pots and 12 experimental plots per species and per MP type.

Three independent trays with pots were prepared (Figure 10), each one consisting of 2 control pots, 4 pots with 27–32 µm MPs per plant, and four pots with 75–90 µm MPs per plant. In total, these included 6 control pots and 12 experimental pots per species and per MP type. They were kept under controlled greenhouse conditions: air temperature 22–28 °C ± 3 °C and relative air humidity 45–65% ± 10% for 50 days (27 March–16 May 2025). All pots were periodically watered to maintain a 60% water-holding capacity throughout the experiment.

### 4.3. Observations on the Bioaccumulation of MPs in Plants

To explore the bioaccumulation of fluorescent MPs in maize and wheat, thin longitudinal slices of fresh plant material (lateral and central roots, stems and leaves) were prepared at the end of the experiment following a standardized protocol. One drop of distilled water was placed in the center of a glass microscope slide, one plant slice was placed on it, and a clear coverslip was placed on top, avoiding air bubbles. All observations and measurements on the plant material were made with a Leica DM 2000 LED fluorescence microscope (Leica Biosystems). Photographs of the microscopic preparations were taken with a digital camera adapted to the microscope (Leica MC170 HD). The criterion for MP calculation was applied to an overall reading area within 1 cm^2^, examining 20 fields per plant organ from each plant. The calculation (items/cm^2^) assumes the hypothesis of a random distribution of the MP particles. Hence, the number N of the particles sampled on a given surface follows a Poisson distribution [66].

### 4.4. Assessment of the Effect on Plant Growth

Shoot growth was measured periodically during the experiment and again at the end of the biotest, when more detailed measurements of the length and biomass of the underground and aboveground parts of each plant for each experimental treatment and for the control were performed. Length was measured using a standard ruler. Dry biomasses were recorded after drying at 70 °C to a constant weight using an analytical balance Model AMB-BA-def-1636, KERN, Balingen, Germany.

### 4.5. Assessment of the Effect on Plant Physiology

The intensity of photosynthesis and transpiration, as well as the content of photosynthetic pigments in the leaves, were measured using standardized methods at the end of the experiment. Pigment extraction was performed by adding 10 mL of 90% acetone to 0.1 g of plant material ground in a porcelain mortar. After filtration, absorbance was measured on a spectrophotometer at 440.5 nm for carotenoids, 662 nm for chlorophyll a, and 644 nm for chlorophyll b. All analyses were performed in triplicate [67]. Measurements of the intensity of photosynthesis, transpiration, and stomatal conductance were performed using a portable photosynthetic system Q-box CO650 (Qubit Systems Inc., Kingston, Canada). Physiological measurements were performed on three fully developed, undamaged leaves of the same physiological age, with at least 30 values taken for each leaf [68].

### 4.6. Statistical Processing

All results were processed using SPSS 19 ver. The significance level at which the null hypothesis was rejected was *p* < 0.05. Values from observations were recorded for each plant and were then averaged for each pot. All errors are indicated as standard deviations. The data were screened for normal distribution using q-q plots and Shapiro–Wilk tests and homogeneity of variance using Levene’s test. Comparisons among treatments were performed by two independent one-way ANOVAs, followed by Tukey’s HSD test at *p* < 0.05.

## 5. Conclusions

Based on the bioassays conducted with fluorescent polyethylene microplastics and the analysis of the results obtained, the following conclusions can be drawn:

(1) The germination process of maize and wheat seeds is not affected by the presence of the tested types of microplastics in the soil.
(2) A statistically significant impact of microplastics was found in terms of the growth and synthesis of biomass, both in underground and aboveground organs, with the degree of impact depending on the type of plant and the size of the plastic particles.
(3) Polyethylene microplastics affect the biosynthesis of photosynthetic pigments, and hence the intensity of the photosynthesis process, with the effect being positive in maize and negative in wheat.
(4) The penetration and accumulation of microplastics from the two studied types (27–32 μm and 75–90 μm) in all organs of the test plants have been observed—roots, stem, leaves.

Biotests using fluorescent microplastics are a quick and reliable way to show whether different plant species can absorb MPs from the soil through the root system and translocate them from underground to aboveground organs. Penetrating the plant organism, MPs have a significant impact on plants, disrupting one of the fundamental processes—photosynthesis, responsible for the synthesis of the compounds necessary for their existence. Particle size is an important factor in their ability to bioaccumulate and in their impact on plants’ vital processes.

## Figures and Tables

**Table 1:** Relative Growth Rate (RGR) of maize and wheat under MPs exposure (mean ± SD).

Crop	Treatment	RGR (Day^−1^)
Maize	Control	0.039 ± 0.0023
	75–90 µm PE	0.034 ± 0.0025
	27–32 µm PE	0.026 ± 0.0017
Wheat	Control	0.024 ± 0.0011
	75–90 µm PE	0.016 ± 0.0001
	27–32 µm PE	0.024 ± 0.0021

**Table 2:** Biometric measurements of maize and wheat plants at the end of the experiment with MPs (mean ± SD). Different letters (small for maize and capital for wheat) indicate statistically significant differences at *p* ≤ 0.05.

Crop	Treatment	Shoot Length (cm)	Root Length (cm)	Shoot Weight (g)	Root Weight (g)
Maize	Control	31.65 ± 1.2 a	16.75 ± 0.9 b	2.67 ± 0.1 b	3.85 ± 0.3 b
	75–90 µm PE	34.95 ± 1.3 a	20.0 ± 1.0 c	2.14 ± 0.1 a	1.30 ± 0.08 a
	27–32 µm PE	30.65 ± 1.2 a	10.20 ± 0.4 a	2.52 ± 0.1 ab	2.06 ± 0.1 a
Wheat	Control	25.85 ± 1.6 A	11.75 ± 0.8 A	0.40 ± 0.2 B	0.18 ± 0.02 B
	75–90 µm PE	29.45 ± 1.8 B	18.8 ± 1.3 B	0.26 ± 0.1 A	0.15 ± 0.01 A
	27–32 µm PE	30.2 ± 2.0 B	12.0 ± 1.1 A	0.27 ± 0.1 A	0.26 ± 0.01 C

**Table 3:** Physicochemical properties of soil substrate after the 50-day experiment with MPs (mean ± SD).

Parameter	Experimental Treatment					
	Maize		Wheat			
	Control	75–90 µm PE	27–32 µm PE	Control	75–90 µm PE	27–32 µm PE
Total nitrogen (Total N), mg kg^−1^	22.17 ± 1.3	20.53 ± 1.0	20.89 ± 1.1	23.07 ± 1.2	22.19 ± 1.6	19.92 ± 1.1
Total phosphorous (Total P), mg kg^−1^	27.43 ± 1.4	28.77 ± 1.2	29.21 ± 1.8	28.85 ± 1.5	28.99 ± 1.4	29.46 ± 1.8
Total potassium (Total K), mg kg^−1^	26.62 ± 1.2	26.13 ± 1.3	26.21 ± 1.3	27.80 ± 1.6	27.72 ± 1.2	26.82 ± 1.8
Organic matter (OM), g kg^−1^	361 ± 26	363 ± 18	363 ± 17	366 ± 21	365 ± 19	362 ± 19
Conductivity, μS·cm^−1^	98.3 ± 7.2	101.1 ± 11.7	97.4 ± 8.1	98.6 ± 9.0	99.2 ± 8.9	95.2 ± 6.6
pH	6.0 ± 0.2	5.9 ± 0.1	5.7 ± 0.2	6.1 ± 0.2	5.7 ± 0.1	5.6 ± 0.2

**Table 4:** Evidence for the effects of MPs on plants.

Parameter	Plant Species	Type of MPS	Size of MPs	Concentration of MPs	Effect of MPs	Reference
Seed germination	*Zea mays*	PE	212–300 μm	0.1% *w*/*w*	null	[48]
	*Zea mays*	PVC	75–150 μm	0.02 g/150 g soil	negative	[50]
	*Triticum aestivum*	PP, HDPE, PLA	150, 1000, 4000 µm	0.01%, 0.05%, 0.1% *w*/*w*	null to negative	[37]
	*Zea mays*	PE	27–32 µm 75–90 µm	0.01% *w*/*w*	null	Present study
	*Triticum aestivum*	PE	27–32 µm 75–90 µm	0.01% *w*/*w*	null	Present study
Shoot length	*Triticum aestivum*	LDPE	200–250 μm	0.5%, 1%, 2%, 5%, 8%	negative	[52]
	*Zea mays*	PE	27–32 µm 75-90 µm	0.01% *w*/*w*	positive	Present study
	*Triticum aestivum*	PE	27–32 µm 75–90 µm	0.01% *w*/*w*	positive	Present study
Shoot biomass	*Zea mays*	Polyurethane-coated fertilizer	3.79 mm	0.01%, 0.1% and 1% *w*/*w*	positive	[47]
	*Triticum aestivum*	PVC and PE	125 μm	1%, 5%, 10% and 20%	null	[53]
	*Triticum aestivum*	LDPE	200–250 μm	0.5%, 1%, 2%, 5%, 8%	positive	[52]
	*Triticum aestivum*	Biodegradable plastics	50 μm, 250 μm, 500 μm	1% *w*/*w*	negative	[51]
	*Triticum aestivum*	PE	200 μm	0.2% *w*/*w*	negative	[14]
	*Triticum aestivum*	PE and PVC	125 μm	1% and 5% *w*/*w*	positive	[54]
	*Zea mays*	PE	27–32 µm 75–90 µm	0.01% *w*/*w*	negative	Present study
	*Triticum aestivum*	PE	27–32 µm 75–90 µm	0.01% *w*/*w*	negative	Present study
Root length	*Zea mays*	PE	212–300 μm	0.1% *w*/*w*	negative	[48]
	*Triticum aestivum*	LDPE	200-250 μm	0.5%, 1%, 2%, 5%, 8%	positive	[52]
	*Zea mays*	PE	27–32 µm 75–90 µm	0.01% *w*/*w*	positive	Present study
	*Triticum aestivum*	PE	27–32 µm 75–90 µm	0.01% *w*/*w*	positive	Present study
Root biomass	*Zea mays*	Polyurethane-coated fertilizer	3.79 mm	0.01%, 0.1% and 1% *w*/*w*	null	[47]
	*Triticum aestivum*	PVC and PE	125 μm	1%, 5%, 10% and 20%	null	[53]
	*Triticum aestivum*	LDPE	200–250 μm	0.5%, 1%, 2%, 5%, 8%	positive	[52]
	*Triticum aestivum*	Biodegradable plastics	50 μm, 250 μm, 500 μm	1% *w*/*w*	negative	[51]
	*Triticum aestivum*	PE	200 μm	0.2% *w*/*w*	negative	[14]
	*Triticum aestivum*	PE and PVC	125 μm	1% and 5% *w*/*w*	positive	[54]
	*Zea mays*	PE	27–32 µm 75–90 µm	0.01% *w*/*w*	negative	Present study
	*Triticum aestivum*	PE	27–32 µm 75-90 µm	0.01% *w*/*w*	negative	Present study
Chlorophyll content	*Zea mays*	PVC	75-150 μm	0.02 g/150 g soil	negative	[50]
	*Zea mays*	Polyurethane-coated fertilizer	3.79 mm	0.01%, 0.1% and 1% *w*/*w*	positive	[47]
	*Triticum aestivum*	Biodegradable plastics	50 μm, 250 μm, 500 μm	1% *w*/*w*	null	[51]
	*Triticum aestivum*	PE	200 μm	0.2% *w*/*w*	positive	[14]
	*Zea mays*	PE	27–32 µm 75–90 µm	0.01% *w*/*w*	positive negative	Present study
	*Triticum aestivum*	PE	27–32 µm 75–90 µm	0.01% *w*/*w*	negative	Present study

**Table 5:** Main characteristics of the substrate used before the experiment.

Parameter	Value
Total nitrogen (Total N)	24.65 mg kg^−1^
Total phosphorus (Total P)	31.90 mg kg^−1^
Total potassium (Total K)	27.55 mg kg^−1^
Organic matter (OM)	370 g kg^−1^
pH	6.1
Conductivity	107.2 μS·cm^−1^
Humidity	64%

## Data Availability

The original contributions presented in this study are included in the article material. Further inquiries can be directed to the corresponding author.
